# Small Molecules Dorsomorphin and LDN-193189 Inhibit Myostatin/GDF8 Signaling and Promote Functional Myoblast Differentiation*

**DOI:** 10.1074/jbc.M114.604397

**Published:** 2014-11-03

**Authors:** Daniel Horbelt, Jan H. Boergermann, Apirat Chaikuad, Ivan Alfano, Eleanor Williams, Ilya Lukonin, Tobias Timmel, Alex N. Bullock, Petra Knaus

**Affiliations:** From the ‡ Institute for Chemistry-Biochemistry, Freie Universität Berlin, 14195 Berlin, Germany,; the § Structural Genomics Consortium, University of Oxford, Oxford OX3 7DQ, United Kingdom, and; the ¶ Muscle Research Unit, Experimental and Clinical Research Center, 13125 Berlin, Germany

**Keywords:** Bone Morphogenetic Protein (BMP), Myogenesis, Myostatin, Serine/Threonine Protein Kinase, Small Molecule

## Abstract

GDF8, or myostatin, is a member of the TGF-β superfamily of secreted polypeptide growth factors. GDF8 is a potent negative regulator of myogenesis both *in vivo* and *in vitro*. We found that GDF8 signaling was inhibited by the small molecule ATP competitive inhibitors dorsomorphin and LDN-193189. These compounds were previously shown to be potent inhibitors of BMP signaling by binding to the BMP type I receptors ALK1/2/3/6. We present the crystal structure of the type II receptor ActRIIA with dorsomorphin and demonstrate that dorsomorphin or LDN-193189 target GDF8 induced Smad2/3 signaling and repression of myogenic transcription factors. As a result, both inhibitors rescued myogenesis in myoblasts treated with GDF8. As revealed by quantitative live cell microscopy, treatment with dorsomorphin or LDN-193189 promoted the contractile activity of myotubular networks *in vitro*. We therefore suggest these inhibitors as suitable tools to promote functional myogenesis.

## Teaser

**Background:** GDF8/myostatin suppresses myogenic differentiation.

**Results:** The small molecule inhibitors dorsomorphin and LDN-193189 bind to and inhibit the GDF8 receptor ActRII and ALK4.

**Conclusion:** Dorsomorphin and LDN-193189 promote myogenesis *in vitro*.

**Significance:** Detailed molecular characterization of small molecule inhibitors targeting the GDF8/myostatin pathway demonstrates their potential and risk when applied to promote muscle development.

## Introduction

Myostatin, or GDF8 (growth and differentiation factor-8), is a 25-kDa dimeric polypeptide growth factor, which belongs to the TGF-β superfamily of extracellular ligands. Members of this family are essential regulators of diverse processes during embryogenesis as well as in adult homeostasis and regenerative processes (1). The major role of GDF8 in mammals appears to be the control of muscle mass. GDF8 knock-out mice feature a strongly increased muscle mass as a result of both muscle cell hyperplasia and hypertrophy (2). Inactivating mutations in the *MSTN* gene are associated with increased skeletal muscle mass in cattle, sheep, canines, and humans (3–8).

GDF8 has been described as a potent negative regulator of myogenesis *in vivo* and *in vitro* and therefore has been a promising therapeutic target in order to promote myogenesis in muscle-wasting conditions, such as age-related atrophy, cachexia, or muscular dystrophies (9). There is, however, increasing evidence that other ligands of the TGF-β superfamily, especially BMPs,^3^ play critical roles in muscle development and homeostasis (10).

Signaling specificity within the 33-member superfamily of ligands is achieved by combinatorial interactions of the respective ligands with cognate receptor complexes consisting of pairs of type I and type II receptors on the cell surface (11). Within this complex, the constitutively active kinase of the type II receptor activates by transphosphorylation the kinase domain of the type I receptor (12). Activated type I receptors then interact with and transphosphorylate receptor-activated Smad proteins (R-Smads), which act as transcription factors to regulate transcription in concert with transcriptional co-activators or co-repressors (13). In parallel to Smad proteins, TGF-β superfamily receptors initiate other signaling pathways that do not directly involve Smads, such as extracellular signal-regulated kinase (ERK), p38 mitogen-associated kinase (p38-MAPK), or Akt pathways, to elicit transcriptional or non-transcriptional responses (14, 15).

Following secretion and activation, GDF8 binds to its type II receptor, either activin type II receptor A or B (ActRIIA or ActRIIB), before a type I receptor, activin-receptor like kinase 4 (ALK4), or -5 (ALK5), is recruited into a heteromeric signaling complex (16, 17) and in turn phosphorylates primarily the TGF-β Smads, Smad2 and Smad3. Both type I and type II receptors feature an extracellular, N-terminal ligand binding domain with a typical three-finger toxin fold (18–21), a single transmembrane domain, and a C-terminal serine/threonine kinase domain (22, 23).

Inhibitors of TGF-β superfamily signaling have been developed mainly by targeting the kinase activity of the type I receptors by ATP-competitive small molecule inhibitors (24, 25). As a consequence of the structural homology of these receptors, there is significant cross-reactivity within the family but also with other kinases, which prompts a more detailed characterization of the inhibitors at hand as well an intensified search for more specific compounds (26).

Although initially identified as Compound C, an inhibitor of AMP-activated protein kinase, dorsomorphin was later recognized for its potential to induce dorsalization in zebrafish embryos and to inhibit BMP Smad- and non-Smad signaling by targeting the BMP type I receptors ALK1, -2, -3, and -6 (27, 28). The dorsomorphin derivative LDN-193189 shares with dorsomorphin the central pyrazolo[1,5-a]pyrimidine moiety and was reported to target the BMP type I receptors with increased potency and specificity (29, 30).

Here, we report that dorsomorphin and LDN-193189 activities, even within the TGF-β family, are not restricted to type I receptors but extend also to the type II receptors ActRIIA and ActRIIB. We report the co-crystal structure of dorsomorphin bound to the receptor ActRIIA. By targeting the type II and type I receptors for GDF8, dorsomorphin and LDN-193189 inhibited antimyogenic GDF8 signaling and were efficient promotors of functional myogenesis *in vitro* in C2C12 cells and primary human skeletal myoblasts.

## EXPERIMENTAL PROCEDURES

### 

#### 

##### Maintenance of Cell Lines

C2C12 cells were obtained from ATCC and maintained in Dulbecco's modified Eagle's medium (DMEM; Biochrom) supplemented with 10% FCS, 2 mm l-glutamine, and 100 units/ml penicillin/streptomycin. Human primary myoblasts were kindly provided by Prof. S. Spuler (Charité-ECRC, Berlin), which had been obtained from healthy donors by muscle biopsies with permission of the local ethics commission (EA 1/203/08) as described previously (31). Cells were maintained in skeletal muscle cell growth medium (PromoCell) supplemented with SupplementMix C-39365 (PromoCell), 20% FCS, 2 mm l-glutamine, and gentamycin.

##### Protein Expression

The kinase domains of human ActRIIA (residues 191–488) and ActRIIB (residues 190–487) were cloned into the vector pFB-LIC-Bse. Baculoviral expression was performed in Sf9 insect cells at 27 °C, shaking at 110 rpm. Cells were harvested at 48 h postinfection and resuspended in 50 mm HEPES, pH 7.5, 500 mm NaCl, 5 mm imidazole, 5% glycerol, supplemented with protease inhibitor set V (Calbiochem). Cells were lysed either using a C5 high pressure homogenizer (Emulsiflex) or by sonication (Sonics Vibra Cell) on ice. Insoluble material was excluded by centrifugation at 21,000 rpm. Nucleic acids were removed either using a DEAE-cellulose column or by the addition of 0.15% polyethyleneimine, pH 7.5, before centrifugation. Proteins were purified using an N-terminal hexahistidine tag by nickel affinity chromatography. The proteins were eluted using 250 mm imidazole in a buffer comprising 50 mm HEPES, 300 mm NaCl, 0.5 mm tris-(2-carboxyethyl)phosphine. The eluted protein was cleaved with tobacco etch virus protease and further purified by size exclusion chromatography using a S200 HiLoad 16/60 Superdex column. A final cleanup step was performed if necessary using reverse purification on a Ni-Sepharose column. The buffer was adjusted to 50 mm HEPES, pH 7.5, 300 mm NaCl, 10 mm DTT, 50 mm l-arginine, and 50 mm l-glutamate for crystallization trials.

##### Crystallization, Data Collection, and Structure Determination

Crystallization was performed using the sitting drop vapor diffusion method at 20 °C. ActRIIA protein buffered in 50 mm Hepes, pH 7.5, 300 mm NaCl, and 0.5 mm tris-(2-carboxyethyl)phosphine was concentrated to 20 mg/ml and mixed with 1 mm dorsomorphin. Viable crystals were obtained in a 150-nl drop mixing the protein and the reservoir solution containing 28% PEG3350, 0.2 m lithium sulfate, 10% ethylene glycol, and 0.1 m Tris-HCl, pH 8.8, at a 1:1 volume ratio. Crystals were cryoprotected with the mother liquor supplemented with 20% ethylene glycol before vitrification in liquid nitrogen. Diffraction data collected at the Diamond Light Source beamline I03 were processed and scaled with MOSFLM (32) and Scala from the CCP4 suite (33), respectively. The structure determination was achieved by molecular replacement using the program Phaser (34) and the coordinates of ActRIIB (23) as a search model. Subsequent manual model building alternated with structure refinement was performed in COOT (35) and Refmac (36), respectively. A TLS model calculated from the TLSMD server (37) was used in the late refinement step. The final model was verified for its geometric correctness with MOLPROBITY (38).

##### Isothermal Titration Calorimetry

Isothermal titration calorimetry experiments were performed at 15 °C using a VP-ITC instrument (Microcal/GE Healthcare). Proteins and ligands were buffered in 50 mm HEPES, pH 7.5, 150 mm NaCl, 2 mm tris-(2-carboxyethyl)phosphine, 2% DMSO. Heats of dilution were determined in a separate experiment and subtracted from the final data. The resulting data were fitted using the non-linear least squares curve fitting algorithm implemented in the Origin software provided with the instrument. Reported errors correspond to those derived from the fitting procedure.

##### Thermal Shift Assay

Thermal melting experiments were performed using a real-time PCR machine, Mx3005p (Stratagene), with a protein concentration of 2 μm and 12.5 μm inhibitor as described previously (39, 40). A kinase-directed compound set was assembled from commercial suppliers, including Biofocus (DPI) and Calbiochem. Screening was performed using a construct of the human ActRIIA protein comprising residues 186–494.

##### Phosphoprotein Assays and Western Blot Analysis for Myogenic Markers

Primary human myoblasts before or during differentiation or C2C12 myoblasts in 12-well plates were starved for 3 h in DMEM, 0.2% FCS, followed by pretreatment with the respective inhibitors, dorsomorphin (Calbiochem), LDN-193189 (Sigma-Aldrich), SB-431542 (Sigma-Aldrich), or DMSO as a vehicle control. Cells were stimulated or not with ligands, rhBMP2 (kind gift by Prof. W. Sebald, Würzburg), rhGDF8 (R&D Systems), recombinant human TGF-β1 (Tebu-Bio), or concentration-matched vehicle control. Cells were lysed directly in SDS sample buffer, and proteins were detected by SDS-PAGE and Western blotting using antibodies for skeletal type II myosin heavy chain (Sigma-Aldrich), phospho-Smad2 (Zymed Laboratories), phospho-Smad1/5 (Cell Signaling), phospho-p38 (Promega), GAPDH (Cell Signaling), or β-tubulin (Sigma-Aldrich) in combination with species-specific HRP-conjugated secondary antibodies (Dianova). Chemoluminescence (Femto-Glo ECL, P.J.K) was recorded using the ChemiSmart5000 digital imaging system (Vilber-Lourmat). Signal intensities were quantified densitometrically on 16-bit raw images using Bio1D software (Vilber-Lourmat).

For the detection of myogenic marker proteins after short term differentiation, confluent C2C12 cells were switched to differentiation medium (DMEM containing 2% horse serum) in the presence or absence of GDF8 and inhibitors as indicated. After direct cell lysis in SDS sample buffer, lysates were analyzed by SDS-PAGE and immunoblotting for the myogenic transcription factors MyoD (Santa Cruz Biotechnology) and myogenin (Santa Cruz Biotechnology). GAPDH (Cell Signaling) served as a loading control.

##### Luciferase Reporter Gene Assays

For Dual-Luciferase assays, C2C12 cells were transfected in 96-well plates with inducible BRE-luciferase or (CAGA)_12_-luciferase reporter gene constructs together with constitutively expressed *Renilla* luciferase (pRLTK-luc) (41, 42). After 16 h, cells were starved for 2 h and stimulated for 6 h with ligand in the presence or absence of kinase inhibitors as described at the indicated concentrations. Luciferase activity was quantified using the Dual-Luciferase reporter gene assay in a Mithras LB940 multimode plate reader (Berthold Technologies).

For Myg-luciferase assays, C2C12 cells were transfected in 96-well plates with the myogenin-luciferase construct (43). At confluence, cells were pretreated with inhibitors for 30 min before stimulation with ligand as indicated or DMSO vehicle control in differentiation medium (DMEM plus 2% horse serum). After 3 days of differentiation, cells were lysed, and firefly luciferase activity was measured as described.

##### Monitoring and Quantification of Myogenic Differentiation by Immunofluorescence Microscopy

To monitor the initial phase of myogenic differentiation *in vitro*, primary human myoblasts or C2C12 cells were grown to confluence in 24- or 12-well plates, respectively. Medium was replaced by differentiation medium (DMEM containing 2% horse serum) with or without inhibitors, human recombinant Noggin (kind gift by Dr. A. Economides, Regeneron Pharma, Tarrytown, NY), or DMSO as well as GDF8 at the indicated concentrations. After 3 days of differentiation, cells were paraformaldehyde-fixed, permeabilized by Triton X-100, and immunostained using antibodies for skeletal type II myosin heavy chain (Sigma-Aldrich). Nuclei were stained with DAPI. Myogenic differentiation was documented using a Zeiss Axiovert 200M epifluorescence microscope and Axiovision (Carl Zeiss) software.

For extended periods of myogenic differentiation, primary human myoblasts or C2C12 cells were grown to confluence in Matrigel-coated or uncoated 24-well plates, respectively, and allowed to undergo myogenic differentiation for 4 days. From day 4 of differentiation, cells were treated with dorsomorphin or DMSO as vehicle control in fresh differentiation medium for another 2 days. After 6 days, myogenic differentiation was visualized as described above.

For high resolution immunofluorescence microscopy of long term differentiation, C2C12 cells were grown to confluence on Matrigel-coated 8-well Permanox multichamber slides before the medium was replaced by differentiation medium. Cells were treated with dorsomorphin in fresh differentiation medium at day 4 of differentiation and again at day 5 before they were processed for immunofluorescence at day 6 as described.

Digital images of myosin heavy chain (MHC) and DAPI channels were quantified using a macro that provided batch operability for the ImageJ distribution Fiji (44). Using an automated procedure for homogeneous data sets, MHC-positive areas were quantified as fractions of the total image area. In addition, using MHC-positive areas to define region of interest masks, the accumulated DAPI signals within these regions of interest were used to quantify the fractions of nuclear staining in MHC-positive areas.

##### Live Cell Monitoring and Quantification of Myotube Twitching Activity

C2C12 cells were grown to confluence in 6-well plates before medium was replaced by differentiation medium. At day 4 of differentiation, the differentiation medium was replaced by differentiation medium containing dorsomorphin, LDN-193189, or DMSO as vehicle control. The contractile twitching of myotubes was recorded at day 6 by live cell differential interference contrast (DIC) microscopy on a Zeiss Axiovert 200M microscope using Axiovision software (Carl Zeiss).

The contractile activity was quantified by analyzing movies of time lapse image series (3.3 frames/s, 86 frames). All image processing steps were performed with the Fiji ImageJ distribution (44). First, we checked all movies for proper image alignment (registration) and unimpaired illumination. Next, we applied a Gaussian smoothing of two pixels to reduce image noise. Then active image areas were visualized by the Image CorrelationJ plugin evaluating the local correlation in predefined regions of two successive frames (45). In the resulting correlation maps, we determined and removed inactive pixels by thresholding each image. The activity at a specific time point is given by the sum of active pixels related to the total pixel number in each frame.

## RESULTS

### 

#### 

##### Dorsomorphin and LDN-193189 Bind ActRIIA Similarly to ALK2

*In vitro* kinase assays have shown that LDN-193189 potently inhibits the kinase activity of ALK4 and ActRIIA with IC_50_ values of 101 and 210 nm, respectively (26). Furthermore, KINOMEscan (DiscoveRx) profiling of LDN-193189 against 451 kinases has identified ActRIIA and ActRIIB as the second and ninth strongest binders after ALK2, with 96 and 88% binding at 100 nm inhibitor concentration, respectively (46). To confirm the significance of these interactions, we screened the ActRII kinases against a large panel of commercially available kinase inhibitors using a thermal shift assay. The top 20 hits for each kinase are reported in Tables 1 and 2. Significantly, LDN-193189 was ranked as the top inhibitor hit for ActRIIA and second for ActRIIB, whereas dorsomorphin was also among the top ranked inhibitors for both kinases. Binding measurements determined by isothermal titration calorimetry (ITC) showed that both compounds had *K_D_* values in the range 14–58 nm (Tables 1 and 2).

**TABLE 1:** **Inhibitor screening for ActRIIA**

Rank	*T_m_* shift	*K_D_*	Inhibitor	Supplier	Supplier ID
	° *C*				
1	10.67	14 nm	LDN-193189	Paul Yu, Harvard	LDN-193189
2	9.75		K02288	BioFocus	382_0087_0284
3	9.18		Cdk1/2 inhibitor III	Calbiochem (EMD)	217714
4	9.15		Alsterpaullone, 2-cyanoethyl	Calbiochem (EMD)	126871
5	8.45	58 nm	Dorsomorphin	Tocris	3093
6	8.34		GSK inhibitor XII	Calbiochem (EMD)	361554
7	8.04		NSC-664704	Calbiochem (EMD)	422000
8	7.88		K252a	Calbiochem (EMD)	420298
9	6.48		Indirubin E804	Calbiochem (EMD)	402081
10	6.2		Cdc7/CDK9 inhibitor	Calbiochem (EMD)	217707
11	5.93		Quercetin	Calbiochem (EMD)	551600
12	5.72		Aloisine A	Calbiochem (EMD)	128125
13	5.6		NSC-664704	Calbiochem (EMD)	422000
14	5.5		Apigenin	Calbiochem (EMD)	178278
15	5.03		Indirubin-3 monoxime	Calbiochem (EMD)	402085
16	4.98		JNK inhibitor V	Calbiochem (EMD)	420129
17	4.68		IKK inhibitor VII	Calbiochem (EMD)	401486
18	4.19		1-Na-PP1	Calbiochem (EMD)	529579
19	4.15		TGFβ RI Inhibitor IV	Calbiochem (EMD)	616454
20	4.12		Aurora/Cdk inhibitor	Calbiochem (EMD)	189406

**TABLE 2:** **Inhibitor screening for ActRIIB**

Rank	T*_m_* shift	*K_D_*	Inhibitor	Supplier	Supplier ID
	°*C*				
1	15.52		GSK inhibitor XII	Calbiochem (EMD)	361554
2	14.6	ND*^a^*	LDN-193189	Paul Yu (Harvard)	LDN-193189
3	12.22		K02288	BioFocus	382_0087_0284
4	11.17		NSC-664704	Calbiochem (EMD)	422000
5	10.87		TGFβ RI Inhibitor IV	Calbiochem (EMD)	616454
6	10.68		Alsterpaullone, 2-cyanoethyl	Calbiochem (EMD)	126871
7	10.18		LY 364947	Calbiochem (EMD)	2718
8	9.74		Quercetin	Calbiochem (EMD)	551600
9	8.91		K252a	Calbiochem (EMD)	420298
10	8.57		Cdc7/CDK9 inhibitor	Calbiochem (EMD)	217707
11	8.21		Apigenin	Calbiochem (EMD)	178278
12	8.14		WHI-P180	Calbiochem (EMD)	681500
13	7.99	30 nm	Dorsomorphin	Tocris	3093
14	6.6		Indirubin E804	Calbiochem (EMD)	402081
15	6.28		Aloisine A	Calbiochem (EMD)	128125
16	6.17		TGFβ RI Inhibitor IV	Calbiochem (EMD)	616454
17	6.14		JNK inhibitor II	Calbiochem (EMD)	420119
18	5.62		1-Na-PP1	Calbiochem (EMD)	529579
19	5.46		Dasatinib	Sequoia	863127-77-9
20	5.32		TPCA-1	Tocris	TPCA-1

*^a^* Not determined.

**FIGURE 1.**
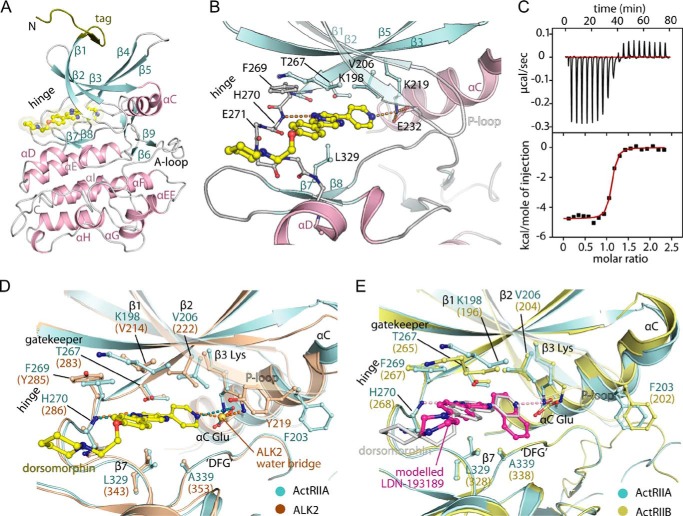
**Inhibitor binding to ActRII.** *A*, overview of the ActRIIA crystal structure showing dorsomorphin bound in the ATP pocket. *B*, interactions of dorsomorphin with ActRIIA. The inhibitor forms direct hydrogen bonds with the kinase domain hinge residue His-270 as well as with the catalytic lysine residue Lys-219, which in turn forms a salt bridge with the catalytic glutamate Glu-232. *C*, binding measurements using ITC show that dorsomorphin binds ActRIIA with a *K_D_* value of 58 nm. *D*, superposition of the ActRIIA and ALK2 (53) structures reveals a similar inhibitor binding pocket. For clarity, only dorsomorphin from the ActRIIA structure is shown. Residue numbers are given for ActRIIA, and any substitutions in ALK2 are shown in *parentheses*. In ActRIIA, the catalytic lysine (Lys-219) forms a bridging hydrogen bonding interaction between dorsomorphin and the catalytic glutamate (Glu-232), whereas in ALK2, the catalytic lysine is displaced, and this interaction is instead mediated by a water molecule. *E*, superposition of the ActRIIA and ActRIIB structures (23) reveals the strict conservation of the residues lining the respective ATP binding pockets. The inhibitor LDN-193189 (*magenta stick representation*) was modeled based on the bound LDN-193189 in ALK2 (26) and the bound dorsomorphin (*gray stick representation*) in this ActRIIA structure. Residue numbers are given for ActRIIA, and any substitutions in ActRIIB are shown in *parentheses*.

Crystallization trials were performed with both dorsomorphin and LDN-193189 to explore the mechanism of inhibition of the type II receptors. Crystals were obtained of ActRIIA with dorsomorphin, from which the complex structure was determined to 2.0 Å resolution with two protein molecules in the asymmetric unit and clear electron density for the bound ligand (see Table 3 for data collection and refinement statistics). The structure represents the first reported of the ActRIIA kinase domain (Fig. 1*A*) and reveals an active conformation with high similarity to that of ActRIIB (root mean square deviation of 0.9 Å over 289 Cα atoms). Differences are restricted to the activation loop, which probably reflects the inherent flexibility of this region.

**TABLE 3:** **Data collection and refinement statistics**

	ActRIIA-dorsomorphin
Protein Data Bank accession code	3Q4T

**Data collection**	
Beamline	Diamond, beamline I03
Wavelength (Å)	0.9763
Resolution (Å)	48.60-1.96 (2.07-1.96)*^a^*
Space group	*P* 6_5_ 2 2
Cell dimensions	*a* = *b* = 110.1, *c* = 206.9 Å
	α = β = 90.0°, γ = 120.0°
No. of unique reflections	53,883 (7,727)
Completeness (%)	99.9 (100.0)
*I*/σ*I*	11.2 (2.3)
*R*_merge_	0.112 (0.783)
Redundancy	7.0 (7.2)

**Refinement**	
Ligands	Dorsomorphin
No. of atoms in refinement (P/L/O)*^b^*	4,899/60/613
*R*_fact_ (%)	16.9
*R*_free_ (%)	22.3
*B_f_* (P/L/O)*^b^* (Å^2^)	30/56/40
Root mean square deviation, bond (Å)	0.016
Root mean square deviation, angle (degrees)	1.5

**Molprobity**	
Ramachandran favored (%)	97.1
Ramachandran allowed (%)	100

*^a^* Values in parentheses show the statistics for the highest resolution shells.

*^b^* P/L/O, protein/ligand molecules presented in the active sites/other (water and solvent molecules).

The high affinity binding of dorsomorphin (Fig. 1, *B* and *C*) is characterized by a single hydrogen bond to the hinge residue His-270 as well as a hydrogen bond from the pendant pyridine to the catalytic β3-strand residue Lys-219, which is also positioned to form the typical salt bridge with the αC residue Glu-232. Overall, the binding mode is highly conserved with that observed in ALK2 (Fig. 1*D*). However, in the inactive ALK2 structure, the salt bridge between the catalytic β3 lysine and αC glutamate is broken, and the pyridine nitrogen of dorsomorphin instead forms a water-mediated hydrogen bond to Glu-248 (αC) (Fig. 1*D*). The binding interactions of ActRIIB are predicted to be similar to ActRIIA, with which the ATP pocket residues are strictly conserved (Fig. 1*E*). Modeling of LDN-193189 is facilitated by the previous co-structure with ALK2 (26). Its interactions are also conserved with dorsomorphin, except for the larger 4-quinoline group, which provides additional hydrophobic interaction (Fig. 1*E*).

##### Dorsomorphin and LDN-193189 Inhibit GDF8-induced Signaling Pathways in Primary Human Myoblasts and in C2C12 Myoblast Precursors

**FIGURE 2.**
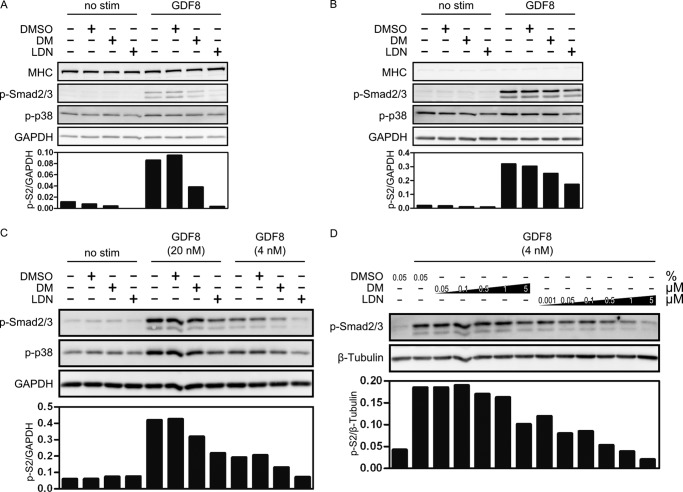
**Dorsomorphin and LDN-193189 inhibit GDF8-induced signaling pathways in undifferentiated and in differentiated primary human myoblasts and in C2C12 premyoblasts.** *A*, primary human myoblasts were allowed to differentiate to myotubes for 6 days, as indicated by expression of MHC. Cells were then serum-starved and pretreated for 30 min with 5 μm dorsomorphin or 0.5 μm LDN-193189, before they were stimulated for 45 min with 8 nm GDF8. GDF8-induced phosphorylation of Smad2/3 and p38 was detected by immunoblotting and quantified densitometrically. *B*, undifferentiated primary human myoblasts were serum-starved, preincubated for 30 min with 5 μm dorsomorphin or 0.5 μm LDN-193189, and stimulated for 45 min with 8 nm GDF8. The absence of MHC expression indicates the undifferentiated status. GDF8-induced phosphorylation of Smad2/3 and p38 was detected by immunoblotting and quantified densitometrically. *C*, serum-starved C2C12 premyoblasts were pretreated for 30 min with 5 μm dorsomorphin or 0.5 μm LDN-193189 before stimulation for 45 min with GDF8. GDF8-induced phosphorylation of Smad2/3 and p38 was detected by immunoblotting and quantified densitometrically. *D*, serum-starved C2C12 premyoblast cells were pretreated for 30 min with increasing concentrations of dorsomorphin or LDN-193189 before stimulation for 45 min with 4 nm GDF8. GDF8-induced phosphorylation of Smad2/3 was detected by immunoblotting and quantified densitometrically.

Based on these results, we evaluated the potential for dorsomorphin and LDN-193189 to inhibit GDF8-induced Smad2/3 phosphorylation in different cell types of the myogenic lineage. Primary human myoblasts were differentiated *in vitro* for 6 days. Pretreatment with 5 μm dorsomorphin or 0.5 μm LDN-193189 interfered with GDF8-induced Smad2/3 phosphorylation (Fig. 2*A*). A similar effect of dorsomorphin and LDN-193189 treatment was observed in undifferentiated primary human myoblasts as well as in the mouse myoblast cell line C2C12 (Fig. 2, *B* and *C*). Repression by LDN-193189 was significantly stronger than by dorsomorphin. In addition, treatment with LDN-193189 also affected GDF8-induced non-Smad signaling, as indicated by reduced p38 MAPK phosphorylation. The relative efficiency of inhibitor treatment was confirmed in more detail in C2C12 cells. Phosphorylation of Smad2/3 was significantly reduced at a concentration of 0.5 μm LDN-193189, whereas 5 μm dorsomorphin was required to achieve the same effect (Fig. 2*D*). The finding that myostatin/GDF8 signaling could be blocked by these putative type I BMP receptor kinase inhibitors in cells of the myogenic lineage led us to investigate the mechanisms of action of dorsomorphin and LDN-193189 inhibition in more detail.

##### Receptor Kinase Inhibitors Exhibit Distinct Specificities for Smad Signaling Induced by TGF-β Family Ligands

**FIGURE 3.**
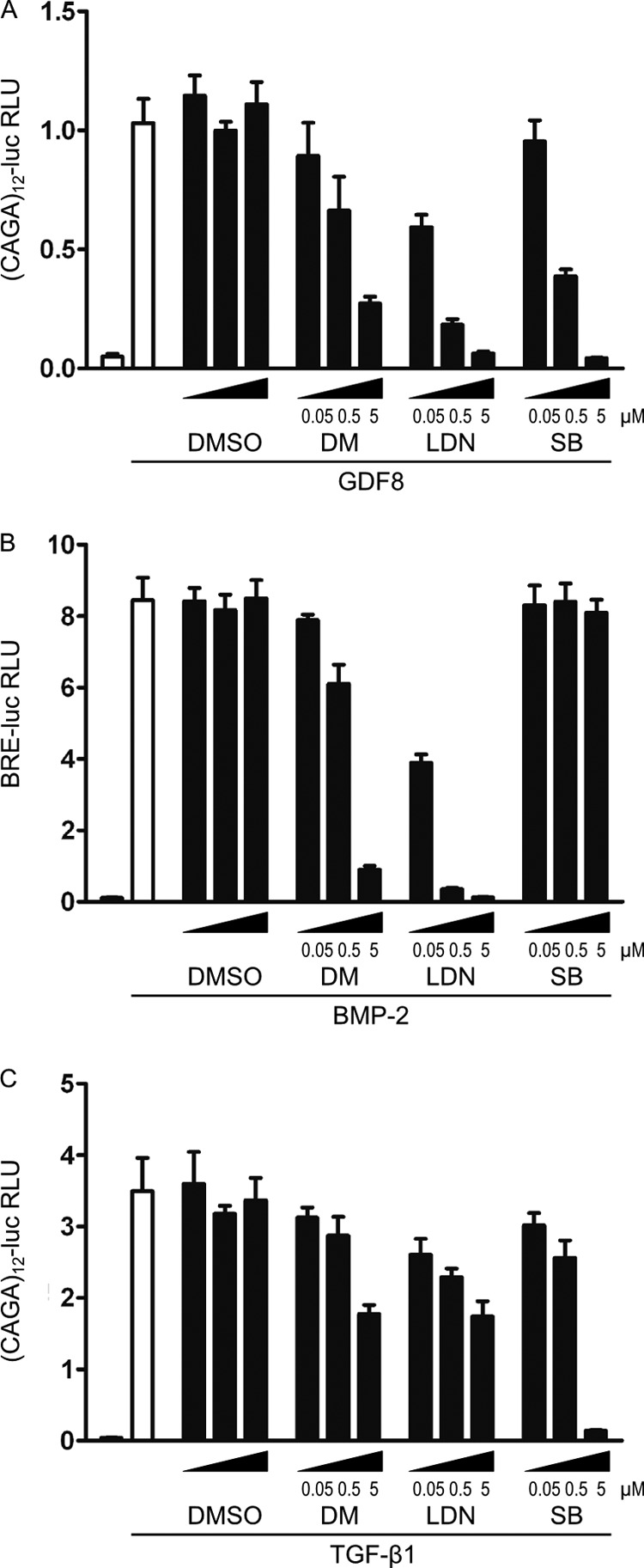
**Dorsomorphin and LDN-193189 efficiently inhibit GDF8 induced Smad3/4 reporter gene activity.** Undifferentiated C2C12 cells were transfected with Smad3/4-responsive (CAGA)_12_-luciferase or Smad1/5-responsive BRE-luciferase reporter constructs together with constitutively expressed *Renilla* luciferase overnight. Cells were serum-starved and stimulated for 6 h with 20 nm GDF8, 100 pm TGF-β, or 10 nm BMP2, together with the receptor kinase inhibitors dorsomorphin, LDN-193189, and SB-431542, as indicated. Luciferase activities are presented as relative luciferase units (*RLU*; firefly luciferase activity normalized to *Renilla* activity). *Bars*, mean ± S.D. (*error bars*) of triplicates. *A*, dorsomorphin and LDN-193189 repressed GDF8-induced (CAGA)_12_-luciferase activity from 0.5 and 0.05 μm, respectively. *B*, BMP2-induced BRE-luciferase activity was efficiently repressed by 0.5 μm dorsomorphin or 0.05 μm LDN-193189. *C*, TGF-β-induced (CAGA)_12_-luciferase activity was repressed by 5 μm dorsomorphin or 0.5 μm LDN-193189.

We next compared the effects of dorsomorphin, LDN-193189, and the ALK4/5/7 inhibitor SB-431542 on the transcriptional activity of GDF8 or TGF-β-induced (CAGA)_12_-luciferase reporter gene activity and on BMP2-stimulated activation of the BRE-luciferase reporter, in C2C12 cells. The (CAGA)_12_-luciferase reporter is activated by the TGF-β R-Smad, Smad3, together with Smad4, whereas BRE is activated by the BMP R-Smads Smad1/5/8 together with Smad4 (41, 42). We found that 5 μm dorsomorphin and 0.5 μm LDN-193189 were sufficient to repress GDF8-induced (CAGA)_12_ -luciferase activity (Fig. 3*A*) by more than 50%. The effect of dorsomorphin was comparable with that of SB-431542, whereas LDN-193189 was even more potent. As expected, the BMP-induced activation of BRE was also efficiently inhibited by 5 μm dorsomorphin and 0.5 μm LDN-193189, whereas SB-431542 had no effect (Fig. 3*B*). By contrast, dorsomorphin and LDN-193189 were less efficient in repressing TGF-β-induced (CAGA)_12_ activation (Fig. 3*C*). Even at 5 μm, dorsomorphin or LDN-193189 did not abolish TGF-β induced (CAGA)_12_-luciferase activation, unlike SB-431542.

**FIGURE 4.**
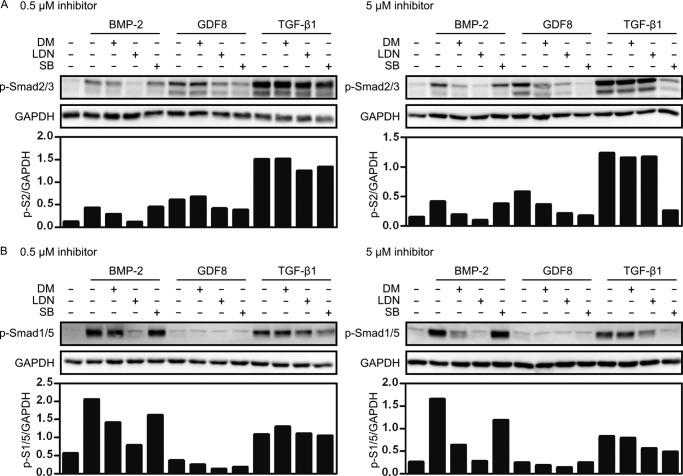
**Ligand-specific effects of kinase inhibitors on Smad2/3 and Smad1/5 phosphorylation.** Serum-starved C2C12 cells were pretreated for 30 min with 0.5 μm (*left panels*) or 5 μm (*right panels*) dorsomorphin, LDN-193189, or SB-431542, before stimulation for 45 min with 5 nm BMP2, 8 nm GDF8, or 100 pm TGF-β1. Phosphorylated Smad2/3 (*A*) or Smad1/5 (*B*) was detected by immunoblotting and quantified densitometrically.

The specific effects on ligand-induced Smad transcriptional activity were confirmed by monitoring Smad phosphorylation. Pretreatment with dorsomorphin or LDN-193189 led to reduced levels of phosphorylated Smad2/3 following GDF8 stimulation (Fig. 4*A*), which were comparable with their effects on BMP2-induced phosphorylation of Smad1/5 (Fig. 4*B*), whereas there was no effect on TGF-β induced Smad2/3 signals (Fig. 4*A*). Taken together, in addition to their known effect on BMP2-induced transcription, we could confirm that dorsomorphin and LDN-193189 efficiently inhibited Smad signaling initiated by GDF8 stimulation.

##### Dorsomorphin or LDN-193189 Promotes Myogenesis in Vitro

**FIGURE 5.**
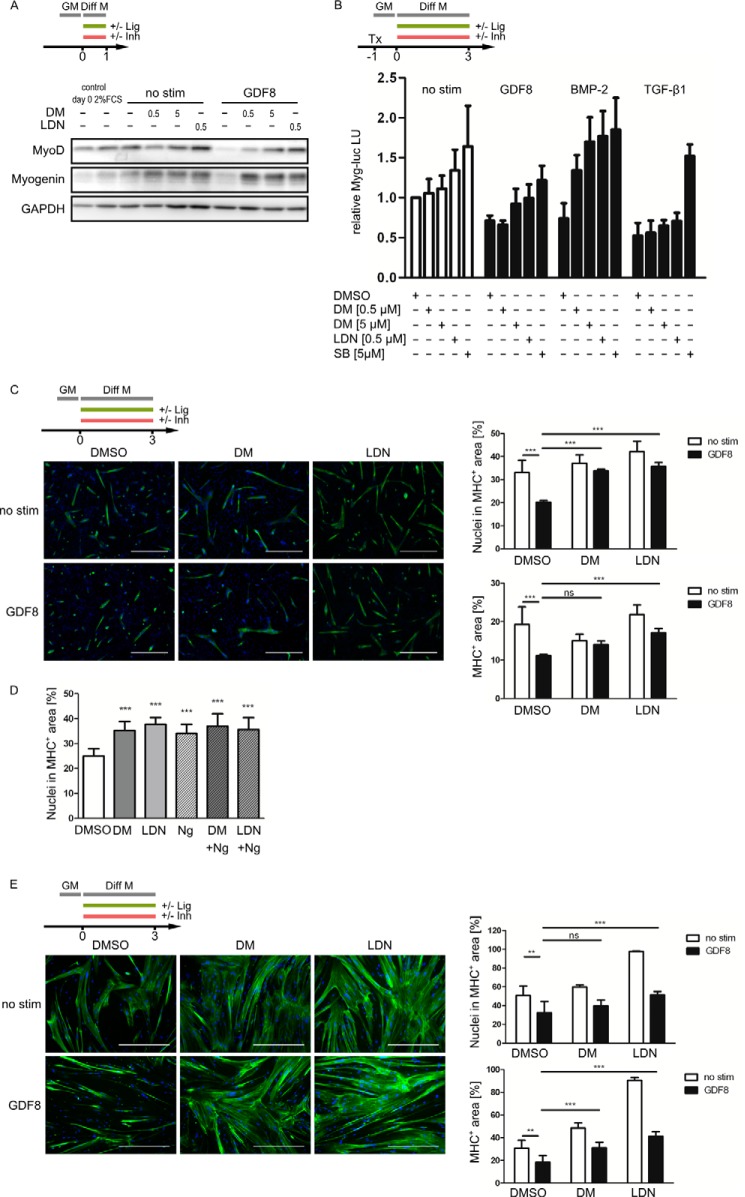
**Dorsomorphin and LDN-193189 counteract GDF8-induced repression of myogenic differentiation.** *A*, confluent C2C12 cells were switched from growth medium (*GM*) to differentiation medium (*Diff M*; DMEM containing 2% horse serum) with inhibitors (*Inh*), dorsomorphin or LDN-193189, and differentiated for 1 day in the presence or absence of ligand (*Lig*), GDF8. Myogenic transcription factors MyoD and myogenin were detected by immunoblotting. The concentration of DMSO was 0.05% in all samples. *B*, C2C12 cells were transfected with a myogenin promoter-dependent firefly luciferase reporter construct (*Myg-luc*) 1 day before the medium was replaced by differentiation medium (DMEM containing 2% horse serum) supplemented with the respective inhibitor. After pretreatment for 30 min, 8 nm GDF8, 5 nm BMP2, or 100 pm TGF-β1 was added. DMSO concentration was 0.05% in all samples. Luciferase activities in lysates were determined after 3 days of differentiation. *Bars*, mean ± S.D. (*error bars*) of Myg-luciferase activities, normalized to unstimulated DMSO controls of four independent experiments. *C*, confluent C2C12 cells were allowed to differentiate for 3 days in differentiation medium (DMEM containing 2% horse serum) in the presence or absence of 5 μm dorsomorphin or 0.5 μm LDN-193189 and stimulated or not with 8 nm GDF8. DMSO concentration was 0.05% in all samples. At day 3, cells were immunostained for skeletal MHC (*green*). Nuclei were stained with DAPI (*blue*). *Scale bars*, 0.5 mm. Myogenic differentiation was quantified using digital image analysis of the fractions of nuclei that were in MHC-positive areas and by the relative MHC-positive area in each picture. *Asterisks* indicate statistical significance (*, *p* < 0.05; **, *p* < 0.01; ***, *p* < 0.001; *ns*, *p* ≥ 0.05) (two-way analysis of variance followed by Bonferroni's multiple comparison test). *D*, confluent C2C12 cells were allowed to differentiate for 3 days in the presence or absence of 5 μm dorsomorphin, 0.5 μm LDN-193189, 4.4 nm noggin, or combinations of those. Myogenic differentiation was quantified as in *C. E*, confluent primary human myoblasts were switched to differentiation medium (DMEM containing 2% horse serum) and pretreated for 30 min with 5 μm DM, 0.5 μm LDN-193189, or DMSO vehicle (0.05%) before stimulation with 8 nm GDF8. After 3 days of differentiation, cells were immunostained for skeletal MHC (*green*). Nuclei were stained with DAPI (*blue*). *Scale bars*, 0.5 mm. Myogenic differentiation was quantified as described for C2C12 cells.

To investigate whether inhibition of GDF8-induced Smad2/3 phosphorylation and transcriptional activity was functionally relevant, myogenic differentiation of C2C12 cells was monitored *in vitro*. As expected, treatment with GDF8 suppressed the up-regulation of myogenic markers MyoD and myogenin (Fig. 5*A*). In the presence of dorsomorphin or LDN-193189, however, this suppressive effect of GDF8 was abolished, and myogenic differentiation was restored.

Besides GDF8, myogenic differentiation of C2C12 cells is known to be attenuated by BMP2 as well as by TGF-β (47–49). Using a luciferase reporter gene under the control of the myogenin promoter (Myg-luc), the expected antimyogenic effects of these growth factors were observed (Fig. 5*B*). Conversely, inhibition of BMP-2 or TGF-β1 signaling by dorsomorphin, LDN-193189, or SB-431542, respectively, promoted Myg-luciferase activity. On the other hand, in cells that were stimulated with GDF8, Myg-luciferase activity was also increased by dorsomorphin or LDN-193189 treatment, although inhibiting ALK5 by SB-431542 was slightly more effective to promote Myg-luciferase activity.

To further explore the potential of dorsomorphin and LDN-193189 to inhibit the antimyogenic effects of GDF8, the initial phase of myogenic differentiation of C2C12 cells was visualized by immunofluorescence microscopy, in which the expression of MHC was visualized and quantified. Images were quantified using ImageJ in a standardized macro-operated procedure regarding the numbers of nuclei that were in MHC-positive areas as well as the total MHC-positive area. Although the presence of GDF8 stimulation strongly suppressed the formation of MHC-positive myotubes after 3 days of differentiation, concomitant treatment with dorsomorphin or LDN-193189 counteracted GDF8, resulting in increased MHC expression (Fig. 5*C*). In cells that were not stimulated with exogenous GDF8, the promyogenic effect of dorsomorphin or LDN-193189 was comparable with that of the BMP antagonist noggin, which was used to scavenge autocrine BMPs (Fig. 5*D*). To investigate whether dorsomorphin and LDN-193189 were also capable of counteracting GDF8 signaling in cells committed to the myogenic lineage, primary human myoblasts were treated with GDF8 in the presence or absence of dorsomorphin or LDN-193189. Similar to C2C12 cells, the formation of myotubes as well as MHC expression were attenuated by GDF8 and could be restored by either dorsomorphin or LDN-193189 (Fig. 5*E*).

**FIGURE 6.**
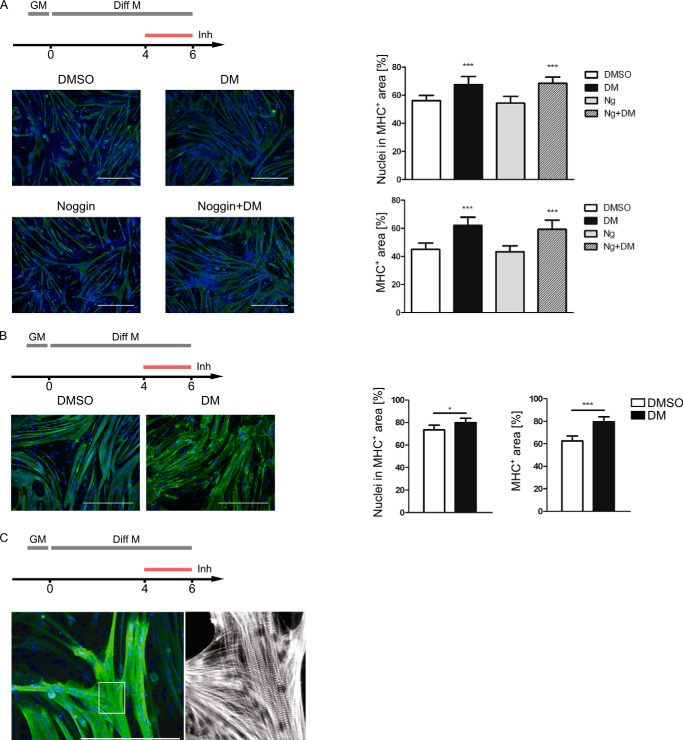
**Dorsomorphin treatment facilitates myotube formation.** *A*, confluent C2C12 cells were switched to differentiation medium (DMEM containing 2% horse serum). At day 4 of differentiation, cells were treated with 5 μm dorsomorphin (*DM*), 4.4 nm noggin, both in combination, or with vehicle (0.05% DMSO). At day 6, cells were immunostained for skeletal MHC (*green*). Nuclei were stained with DAPI (*blue*). *Scale bars*, 0.5 mm. Myogenic differentiation was quantified as in Fig. 5. *B*, confluent primary human myoblasts on Matrigel-coated Permanox slides were switched to differentiation medium (DMEM containing 2% horse serum). At day 4 of differentiation, cells were treated with 5 μm dorsomorphin or vehicle (0.05% DMSO). At day 6, myogenesis was visualized and quantified as in Fig. 5. *C*, dorsomorphin-treated C2C12 cells displayed a typical intermittent MHC staining of A-bands and unstained Z-discs. Confluent C2C12 cells were differentiated for 4 days in differentiation medium (DMEM containing 2% horse serum). At days 4 and 5 of differentiation, cells were treated with 5 μm dorsomorphin. At day 6, cells were immunostained for skeletal MHC (*green*). Nuclei were stained with DAPI (*blue*). *Scale bar*, 0.5 mm. *Right panel*, *enlarged area* of the MHC displayed in *black and white* to depict the striated muscle phenotype.

The positive effects of dorsomorphin and LDN-193189 on myotube formation raised the question whether a similar promyogenic effect was true for later stages of myogenic differentiation. To this end, C2C12 cells or primary human myoblasts were allowed to differentiate for 4 days before they were treated with dorsomorphin for the following 2 days. In addition, the effects of an extracellular BMP antagonist, noggin, were evaluated in C2C12 cells. At day 6 of differentiation, the formation of a myotubular network was visualized by MHC immunocytochemistry. In both cell types, dorsomorphin treatment resulted in increased numbers of nuclei that were in MHC-positive areas and of the total MHC-positive area (Fig. 6, *A* and *B*). Of note, at this stage, noggin alone did not promote myogenesis; nor did noggin further enhance myogenesis in dorsomorphin-treated cells. The myotubular networks that formed in the presence of dorsomorphin revealed the typical morphology and internal organization of contractile myotubes, as represented by intermittent MHC staining of A-bands (Fig. 6*C*) (50).

**FIGURE 7.**
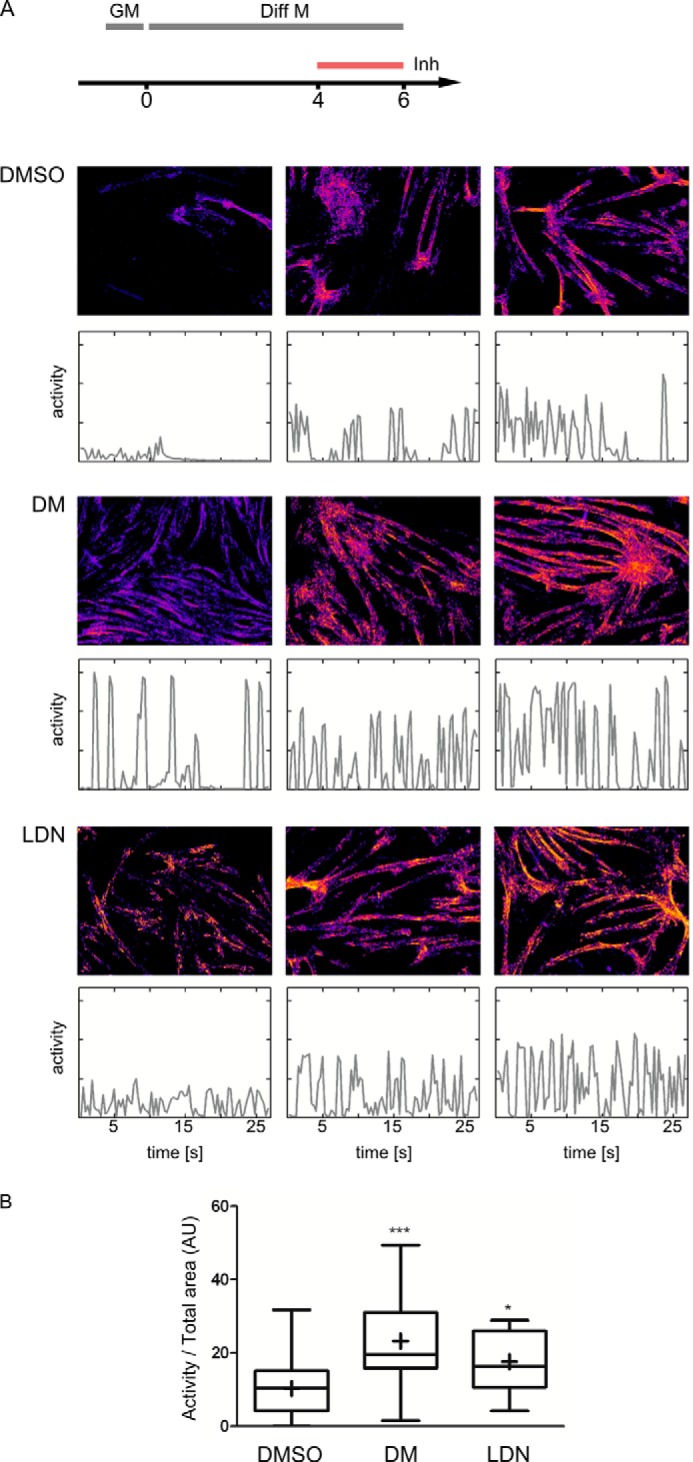
**Dorsomorphin and LDN-193189 promote the formation of a contractile myotube network.** Confluent C2C12 cells were switched to differentiation medium (DMEM containing 2% horse serum). At day 4 of differentiation, cells were treated with 5 μm DM, 0.5 μm LDN-193189, or vehicle (0.05% DMSO). At day 6, contractile activities were recorded by time lapse DIC microscopy (3.3 frames/s, 30 s). *A*, visualization of contractile areas and activities. *Top panels*, contracting areas with *color coding* for the mean activities. Only active areas are *visible*, whereas inactive areas appear *black. Bottom panels*, time course of activity for the respective movies. For each treatment, three representative data sets are shown: samples with activities at the lower 25% (*left*), median (*middle*), or 75% percentiles (*right*) of the mean activities of all samples with the respective treatment. *B*, median activities in movies of DMSO (*n* = 28)-, dorsomorphin (*n* = 32)-, and LDN-193189 (*n* = 28)-treated cells. *AU*, arbitrary units. *Asterisks* indicate statistical significance (*, *p* < 0.05; ***, *p* < 0.001) (Kruskal-Wallis test followed by Dunn's multiple comparison test).

To confirm the potential of dorsomorphin and LDN-193189 to promote functional myogenesis *in vitro*, the contractility of myotubular networks formed by C2C12 cells after 6 days of differentiation was recorded by live cell DIC microscopy. Based on the resulting time lapse movies, areas of twitching myotubes were visualized, and the twitching activity was quantified (Fig. 7, *A* and *B*). In line with our findings that dorsomorphin promoted terminal differentiation and the formation of myotubular networks, quantitative analysis revealed an increased contractile activity in response to treatment with dorsomorphin or LDN from day 4 to 6 (Fig. 7*B*).

Taken together, our data demonstrate that both dorsomorphin and LDN-193189 act as potent promoters of myogenic differentiation. Of note, depending on the time of administration, dorsomorphin and LDN-193189 were capable of promoting both initial and terminal phases of muscle cell differentiation.

## DISCUSSION

### 

#### 

##### A New Light on Dorsomorphin and LDN-193189 Specificity

The search for specific inhibitors of TGF-β superfamily pathways in the past decade was primarily focused on targeting the type I receptors (51). This is reasonable with respect to their position in the pathway directly upstream of Smad activation and of non-Smad pathways (27, 28, 30). Moreover, to date, all known activating mutations in receptors of the TGF-β superfamily map to type I receptors, which makes their specific inhibition desirable from a therapeutic point of view (52, 53).

Studies to determine the specificity of small molecule inhibitors (54, 55) of TGF-β superfamily receptors have been performed with immunoprecipitated or recombinant proteins either by using *in vitro* kinase assays or by *in vitro* binding assays, such as ITC or thermal shift determination, as well as by crystal structures. In complementary cell-based assays, either single constitutively active ALK receptors or limited sets of TGF-β or BMP isoforms, in most cases TGF-β1 or BMP-2, -4, -6, or -7, were used to induce Smad2/3 or Smad1/5/8 phosphorylation, respectively, or Smad-dependent reporter gene activation (26, 27, 30, 46).

Here we demonstrate that dorsomorphin and LDN-193189 can also inhibit GDF8 signaling. Both inhibitors could interfere with GDF8-induced phosphorylation and transcriptional activity of Smad2/3 in GDF8-responsive cell types of the myogenic lineage and at different stages of myogenic commitment. The concentrations yielding significant, although incomplete, repression were 5 μm dorsomorphin or 0.5 μm LDN-193189. At the same concentrations, inhibition of BMP2-induced Smad1/5 signaling was nearly complete, whereas TGF-β-induced Smad2/3 signaling was only slightly affected.

These effects are consistent with the reported inhibitor activities using *in vitro* kinase assays. For example, LDN-193189 is notably more active against the GDF8 receptors ALK4 (IC_50_ = 101 nm) and ActRIIA (IC_50_ = 210 nm) compared with ALK5 (IC_50_ = 350 nm), although all of these kinases are inhibited less efficiently than the BMP-specific ALKs (IC_50_ values in the range 0.8–16.7 nm) (26). The strong binding of dorsomorphin to ActRIIA is additionally demonstrated by our co-crystal structure with the ActRIIA kinase domain as well as by our *T_m_* shift and ITC assay data using both ActRIIA and ActRIIB.

Interestingly, the type II TGF-β receptor TβRII is inhibited by LDN-193189 (IC_50_ = 140 nm) in the same concentration range as ActRIIA (56). Thus, the cellular efficacy of this inhibitor against TGF-β signaling is most likely limited by its low potency against ALK5. Currently, there are no available structural models for the TβRII kinase domain, but its ATP pocket is expected to share considerable structural and sequence conservation with both ALK2 and ActRIIA. In particular, the hinge region of TβRII shares a threonine side chain as its gatekeeper residue, a site most frequently associated with inhibitor selectivity in active kinases (57). In contrast, the type II BMP receptor BMPR2 contains a larger methionine side chain as its gatekeeper receptor. This side chain is predicted to sterically interfere with LDN-193189 binding, and indeed a lack of binding has been reported (46).

Thus, we suggest that the pronounced effect of dorsomorphin or LDN-193189 on GDF8-induced signaling may result from the functional synergism of inhibiting both the GDF8 type II (ActRIIA and -B) and type I (Alk4 and Alk5) receptors. Our conclusion raises the possibility of achieving functional specificity with inhibitory compounds that target more than one signaling component with intermediate efficiency rather than being highly specific and efficient for a single component. This concept may inspire the search for strategies to interfere with specific functions of pleiotropic signaling pathways.

Over the years, evidence has accumulated that the separation between TGF-β-induced Smad2/3, on one hand, and BMP-induced Smad1/5/8, on the other hand, may be less stringent than previously thought (54, 58, 59). In these studies, the inhibitors LDN-193189 and SB-431542 have provided useful complementary tools to interrogate these pathways for the respective activities of ALK1/2/3/6 and ALK4/5/7. Our data confirmed that TGF-β stimulation resulted in significant phosphorylation of Smad1/5 in addition to Smad2/3. TGF-β-induced Smad1/5 phosphorylation was suppressed by SB-431542 or LDN-193189, although the effect of LDN-193189 was far weaker. This might suggest that both the BMP-ALKs and ALK5 contributed to this Smad1/5 phosphorylation, either in parallel or cooperatively. Interestingly, GDF8 also induced some weak Smad1/5 phosphorylation, although this was hard to quantify against a background of high Smad1/5 phosphorylation resulting from BMP2 and TGF-β. In our experience, 4 or 20 nm GDF8 was able to induce weak phosphorylation of Smad1/5, which was sensitive to inhibition by dorsomorphin or LDN-193189. Vice versa, we observed phosphorylation of Smad2/3 in response to BMP2 stimulation. This was previously reported by other groups for BMP2 and BMP4 (54, 55). Of note, in our experiments, this BMP2 activity was inhibited by dorsomorphin or LDN-193189 but not by SB-431542, demonstrating that the kinase activities of ALK4/5/7 were not required.

Thus, our data emphasize that there is significant interaction between the receptors for TGF-β and BMPs with both groups of Smads. Taking into account recent findings regarding their effects on various receptors, including type II receptors, dorsomorphin or LDN-193189 may need to be complemented by more specific inhibitors to dissect these interactions.

##### Dorsomorphin and LDN-193189 as Tools to Promote Functional Myogenesis in Vitro

The inhibition of GDF8 signaling that was observed in immediate pathway readouts was physiologically relevant and sufficient to affect the most prominent function of GDF8 signaling, which is the repression of myogenesis. At this point, it should be considered that TGF-β and BMP signaling, which are targeted slightly or very potently, respectively, by dorsomorphin and LDN-193189, can be antimyogenic *in vitro* as well. Whereas TGF-β appears to counteract differentiation in myoblasts through a pro-proliferative effect and through the repression of myogenic transcription factors (60, 61), the action of BMPs is mediated by up-regulation of Id (inhibitor of differentiation) proteins, which competitively inhibit the formation of transcriptionally active dimers of promyogenic transcription factors, such as MyoD and E-box proteins (62). GDF8, in addition to a suggested pro-proliferative effect (61), represses both the activity and transcription of MyoD and myogenin (63–65).

The repression of myogenic transcription factors could be observed immediately in short term GDF8-stimulated C2C12 cells and was significantly counteracted by dorsomorphin and LDN-193189. Using a luciferase reporter gene assay to measure the activity of the myogenin promoter, we saw that dorsomorphin and LDN-193189 were able to enhance myogenin transcription in GDF8-stimulated cells. As expected, both compounds were even more potent against BMP stimulation, whereas TGF-β signaling was only robustly affected by the ALK4/5/7 inhibitor. Of note, SB-431542 appeared to be a strong promoter of myogenin expression irrespective of stimulation with ligand.

To prove the potential of dorsomorphin and LDN-193189 to promote functional myogenesis, cells were treated for longer periods of time. In contrast to direct and immediate effects of dorsomorphin and LDN-193189 after short term treatment, autocrine and serum effects need to be considered in this protocol. In line with previous publications, inhibition of BMP signaling using noggin, dorsomorphin, or LDN-193189 promoted myogenesis in C2C12 cells after 3 days of continuous treatment (49). Of note, the reported effects of dorsomorphin and LDN-193189 were comparable with those of noggin treatment. Despite the fact that in our experiments, the effects of dorsomorphin or LDN-193189 were more pronounced in the presence of exogenous GDF8 as compared with unstimulated cells, we conclude that inhibition of autocrine BMP signaling by dorsomorphin and LDN-193189 at this time point contributed significantly to promote myogenic differentiation. Signaling of endogenous BMPs, however, was reported to decrease rapidly during the course of differentiation between days 2 and 4, whereas the expression of TGF-β isoforms increases (48, 49). Expression of endogenous GDF8 was reported to be present in myotubes but not in undifferentiated C2C12 (66). We thus used a modified protocol, in which C2C12 cells were allowed to differentiate for 4 days before the inhibitors were added in fresh differentiation medium for an additional 2 days. In this protocol, dorsomorphin was still effective to promote myogenic differentiation, whereas the effect of noggin had vanished, indicating that the potential of dorsomorphin to promote late myogenesis was not related to inhibition of one of the BMP isoforms that is bound by noggin (*i.e.* BMP2, -4, or -7). It may, however, still be possible that inhibition of autocrine signaling by other BMP isoforms (*e.g.* BMP6) contributed to the effects of dorsomorphin also at this stage.

At day 6, extended twitching networks of multinucleated myotubes were established. In order to evaluate whether the promyogenic effects of dorsomorphin and LDN-193189 resulted in an increased contractile activity of these networks, we developed an automated procedure for the quantification of time lapse movies. This procedure is based on a comparison of two subsequent frames and has proven very robust against distortions by moving particles, such as cellular debris. By comparing cells that had been treated with dorsomorphin or LDN-193189 at day 4 for an additional 2 days, we could confirm that the beneficial effects of these inhibitors result in enhanced functional myogenesis. We therefore suggest dorsomorphin and LDN-193189 as tools to generate functional myotubular networks, which may be used as an *in vitro* model system. It will be rewarding in the future to characterize these myotubes in more detail.

Overall, we have demonstrated a novel and unexpected utility of dorsomorphin and LDN-193189 as inhibitors of GDF8, as well as of BMP signaling, to promote functional myogenesis *in vitro*. However, it is clear that the use of dorsomorphin or LDN-193189 is not a promising therapeutic option for patients suffering from muscle-wasting diseases, such as Duchenne muscular dystrophy. As reported recently, BMP signaling *in vivo* is a strong promoter of muscle hypertrophy, and inhibition of BMP signaling leads to muscle atrophy (10). Thus, the potent effect of dorsomorphin and LDN-193189 as repressors of BMP signaling may provoke adverse effects.

In general, there is increasing evidence that the beneficial effects of GDF8 inhibition as a therapeutic strategy in muscle-wasting conditions, such as cachexia, age-related atrophy, or muscular dystrophies, need to be evaluated with care. It seems that the hypertrophic phenotype in GDF8-knock-out mice does not confer increased muscle strength and endurance (67, 68). Recently, it was shown that an increased muscle mass was not associated with functional improvements because GDF8 knock-out mice suffered from extreme fatigability and exercise intolerance (69). Of note, similar effects were observed by a blockade of GDF8 signaling in adult mice using the GDF8 propeptide or a soluble ActRIIB-Fc fusion (69, 70). In the latter study, developmental consequences of a lack in GDF8 signaling, which could account for a decreased ratio between oxidative and glycolytic fibers, were not observed (71). Instead, inhibition of GDF8 signaling appeared to have direct metabolic effects on the oxidative capacities of skeletal muscles (70).

Two clinical phase 2 trials using sActRIIB-Fc in Duchenne muscular dystrophy (clinicaltrials.gov identifiers NCT01099761 and NCT01239758) had to be terminated prematurely due to side effects, despite encouraging improvements in muscle mass. In this respect, it is noteworthy that sActRIIB is expected to scavenge not only GDF8, but also BMP-9, -10, and -11 (16, 72). The observed side effects, minor bleeding events and reversible vascular abnormalities, are also potentially attributable to the functions of BMP-9 and -10 in the regulation of angiogenesis (73). On the other hand, circulating BMP-11, also named GDF-11, was recently suggested to be a factor that promotes the functionality of skeletal muscle (74).

In light of the increasing knowledge about the function of TGF-β superfamily signaling pathways in diverse processes of development and homeostasis, the development of innovative strategies for therapeutic intervention seems more challenging than ever. Our study underscores the importance of extensive characterization of potential tools, such as small molecule inhibitors, and it highlights the need to incorporate into this context the concept of achieving functional specificity in contrast to a mere molecular specificity.

## Footnotes

3 The abbreviations used are:

BMP: bone morphogenic protein
DIC: differential interference contrast
MHC: myosin heavy chain
ALK: activin-receptor like kinase.
